# Supplementary material for: Circulatory Inflammatory Mediators in the Prediction of Anti-Tuberculous Drug-Induced Liver Injury Using RUCAM for Causality Assessment
Source: Biomedicines. 2021 Jul 25;9(8):891. doi: 10.3390/biomedicines9080891 (PMC8389605; doi:10.3390/biomedicines9080891)
Supplement: Supplementary file 1 [file biomedicines-09-00891-s001.zip › biomedicines-1323647-supplementary.pdf]

Table S1. Receiver operating characteristic (ROC) curve analysis and area under the ROC curve (AUC) of baseline plasma cytokines in prediction of drug-induced liver injury

	AUC (mean ± SD)	*p*	Cut-off value	Sensitivity	Specificity
FGF-2	0.455 ± 0.065	0.515	-	-	-
IL-10	0.634 ± 0.064	0.053	3.9 pg/mL	0.263	0.964
IL-17A	0.493 ± 0.071	0.923	0.99 pg/mL	0.316	0.738
IL-22	0.562 ± 0.072	0.369	2.4 pg/mL	0.421	0.715
IL-22BP	0.389 ± 0.072	0.110	520 ng/L	0.737	0.100
IL-23	0.527 ± 0.070	0.695	2.1 pg/mL	0.263	0.805
IL-6	0.631 ± 0.068	0.058	19.0 pg/mL	0.421	0.791
IP-10	0.679 ± 0.064	0.010	33.0 pg/mL	0.684	0.620
MIG	0.624 ± 0.065	0.073	53.0 pg/mL	0.842	0.176
MIP-1b	0.626 ± 0.061	0.069	72.6 pg/mL	0.947	0.253
PDGF-BB	0.526 ± 0.067	0.710	0.61 pg/mL	0.842	0.249
RANTES	0.476 ± 0.068	0.729	44.8 pg/mL	1.000	0.032
IL-12p70	0.564 ± 0.072	0.356	1.45 pg/mL	0.368	0.801
CD206	0.603 ± 0.056	0.138	1.147 ng/mL	0.842	0.452
sCD163	0.571 ± 0.074	0.305	1.32 ng/mL	0.789	0.371

Abbreviation: AUC, area under the curve; FGF-2, fibroblast growth factor 2; IL, interleukin; IL-22BP, IL-22 binding protein; IP-10, interferon gamma-induced protein 10; MIG, monokine induced by interferon-gamma; MIP-1b, macrophage inflammatory protein-1beta; PDGF-BB, platelet-derived growth factor-BB; RANTES, Regulated upon Activation, Normal T Cell Expressed and Presumably Secreted; sCD163, soluble CD163; SD, standard deviation
